# Ecological implications of allometric relationships in American alligators (*Alligator mississippiensis*)

**DOI:** 10.1038/s41598-024-56798-5

**Published:** 2024-03-13

**Authors:** Sergio A. Balaguera-Reina, Brittany M. Mason, Laura A. Brandt, Nicole D. Hernandez, Bryna L. Daykin, Kelly R. McCaffrey, Sidney T. Godfrey, Frank J. Mazzotti

**Affiliations:** 1 Department of Wildlife Ecology and Conservation, Fort Lauderdale Research and Education Center, University of Florida, Fort Lauderdale, 33328 USA; 2 U.S. Fish and Wildlife Service, Fort Lauderdale, 33328 USA

**Keywords:** Allometric coefficients, Body condition, Everglades, Management, Morphology, Static allometry, Trait–size relationships, Conservation biology, Evolutionary ecology, Freshwater ecology, Wetlands ecology, Zoology

## Abstract

Morphometric allometry, the effect of size on morphological variation, has been of great interest for evolutionary biologist and is currently used in fields such as wildlife ecology to inform management and conservation. We assessed American alligator (*Alligator mississippiensis*) morphological static allometry across the Greater Everglades ecosystem in South Florida, United States using a robust dataset (~ 22 years) and investigated effects of sex, habitat, and sampling area on morphological relationships. Regression models showed very strong evidence of a linear relationship between variables explaining equal to or above 92% of the variation in the data. Most trait–size relationships (8 out of 11 assessed) showed hyperallometry (positive allometry) with slope deviations from isometry between 0.1 and 0.2 units while the other three relationships were isometric. Sampling area, type of habitat, and in a lesser extent sex influenced allometric coefficients (slope and intercept) across several relationships, likely as result of differing landscapes and ecosystem dynamic alterations and sexual dimorphism. We discuss our findings in terms of the biology of the species as well as the usefulness of our results in the context of ecosystem restoration and conservation of the species. Finally, we provide recommendations when using trait–length relationships to infer population nutritional—health condition and demographics.

## Introduction

Morphometric allometry is an important topic in evolution and development as it describes size-related changes of morphological traits^1^. Organisms across the tree of life vary in size due to developmental, environmental, or evolutionary reasons, adopting shapes that can be predictable based on morphological relationships^2^. Thus, a particular trait is commonly strongly correlated with the size of the whole body following exponential scaling (power-law function *Y* = *aX*^*b*^), where *Y* represents the trait value, *X* represent the body size, and *a* (intercept) and *b* (slope) are parameters describing the relationship^3,4^. Interestingly, when relating two variables that have a linear relationship, *b* can be considered the constant differential growth rate or allometric coefficient, which indicates that the ratio between the rate of growth in *Y* and *X* remains unchanged^4,5^. Thus, the parameters of the allometric equation can be used to summarize trait–size covariation and compare morphologies among populations. Allometric coefficients have proved to be useful for management and conservation purposes, especially those relating weight and length, and have been implemented to assess the effect of environmental factors on the health of individuals and populations^6,7^ and to understand how differences in individual’s weight given a length can be influenced by landscape transformation^8–10^.

Crocodylians have been used as indicators of ecological restoration in wetlands and coastal ecosystems^11^ because of their response to ecosystem change^12^. One important metric considered is body condition, which provides information on the nutritional and health condition of an individual^13^. This metric commonly relates individuals’ weight and length measurements using the slope of the relationship (estimated log-transforming the data) as an exponent of the latter, which is no more than the allometric equation solved for the intercept (allometric elevation *a* = *Y*/*X*^*b*^). Higher values of this ratio mean animals are proportionally heavier and therefore in better condition; lower values mean animals are proportionally lighter and therefore in lower condition^7^. Crocodylian management often requires the ability to quantify animals’ sizes to define population structure^14^, which is normally achieved by visually estimating component parts of animals in the field (i.e., total length—TL extrapolated from head length—HL^15^). This is because demographic and reproductive variables in crocodylians are mostly dependent on body size rather than age, so population analyses are commonly based on the former^14,16^. These two examples show the relevance for crocodylian management of understanding morphometric relationships as well as effects of intrinsic (i.e., sex) and extrinsic (i.e., sampling area and habitat) variables on regression parameters.

Studies focused on understanding morphometric relationships in crocodylians have been done in several species across the world (*Crocodylus acutus*^14^, *C. johnstoni*^15^, *C. porosus*^17^, *C. novaeguineae*^18^, *C. niloticus*^19^, C. *moreletii*^20^) including American alligators (*Alligator mississippiensis*)^21–23^. However, these studies used arithmetic rather than log-transformed data to estimate allometric coefficients, which assumes additive rather than multiplicative errors and absolute rather than relative relationships between variables, which is commonly seen as bias in allometry studies^24^. The bias is because additive error model assumes that equivalent deviations differ by equal amounts whereas multiplicative error model assumes that they differ by equal proportions^24^. Dodson^25^ presented the only up to date allometric study on alligators using log-transformed data. He assessed cranial and skeleton variation, providing insights about functional and ecological significance of relative growth in alligators. Remarkably, there have not yet been studies on live alligators covering a suite of parameters across a large ecosystem assessing the effect of intrinsic (i.e., sex) and extrinsic (i.e., sampling area and habitat) factors in allometric relationships as well as the ecological implications.

Morphological allometry is a relevant topic in biology due to its evolutionary conservatism mainly when referring to the static allometric slope^2,26^. Although two main hypotheses have been postulated to explain this evolutionary stasis (stabilizing selection and absolute genetic constraint)^2,27,28^, recent studies in moths^29^ and fruit flies’ artificial selection (*Drosophila*)^30,31^ provided evidence that supports a combine effect of multivariate constraints (e.g., pleiotropic genes) and multivariate stabilizing selection (hybrid hypothesis)^2^. Interestingly, once artificial selection is relaxed slope-selected populations raced back toward the starting allometry while intercept-selected populations returned very slowly. Thus, an allometric slope in wild populations would lie along a ridge of high fitness whereas allometry elevation (intercept) has more room for change.

In the present study we assessed the static morphological allometry of American alligators in the Greater Everglades ecosystem in South Florida, United States, based on a set of traits (HL, neck girth -NG, chest girth -CG, tail girth -TG, Snout-Vent Length—SVL, and Weight—W) and size (SVL and TL) metrics collected through 22 years (1999–2022). We also investigated whether intrinsic variables such as sex or extrinsic variables such as habitat and sampling area have an effect on trait–size allometric parameters (slope and intercept). We discuss the relevance of our findings in terms of the biology of the species, the implications of the variation of regression parameters at the ecosystem level, and the usefulness of this information within the context of management and conservation. We expect that due to allometry conservatism most of the traits–size relationships won’t be affected by sex, habitat, or sampling area. However, trait–size relationships such as W–SVL and W–TL linked with prey intake could show both intercept (allometry elevation) and in a lesser extent slope (allometry coefficient) variation as it can potentially be affected by prey quality and prey availability.

## Methods

We captured and collected morphometric data from 5508 alligators in the Greater Everglades ecosystem between October 1999 and November 2022. Not all attributes were measured on all alligators because of changes in protocols across various studies. Alligators were captured across six sampling areas (Arthur R. Marshall Loxahatchee National Wildlife Refuge -LOX, water conservation areas -WCA 2, 3A, and 3B, Big Cypress Natural Reserve -BICY, and Everglades National Park -ENP). These sampling areas are well-defined areas with four of them (LOX, WCA2, 3A, and 3B) established as Water Conservation Areas surrounded by manmade levees and canals and managed under a water regulation schedule for water supply, flood protection, and wildlife. Research was conducted under animal research protocols approved by the University of Florida Institutional Animal Care and Use Committee (IACUC 202200000026) in compliance with the ARRIVE guidelines^32^ and performed in accordance with relevant guidelines and regulations. Sampling areas consisted of either marsh, canal, or river habitat. Sampling effort across areas varied because funding and survey protocols^33^. However, our large sample size (5000+ animals) provides a good spatial representation of the alligators in the Greater Everglades.

Surveys were conducted at night using airboats or motorboats to search for alligators with the aid of Fenix HP15 900-lm headlamps and handheld Brinkman Q-beam 200,000 candlepower spotlight (Brinkman, Dallas, TX, USA; 1999–2019) or Fenix TK41C 1,000-lm tactical flashlight (Fenix Lighting, Littleton, CO, USA; 2019–2022). Once animals were identified, we captured them by hand, tong, or snare, marked them via a Passive Integrated Transponder (PIT) tag, toe tag, or scute clipping (or a combination of those), measured them (HL, SVL, TL, NG, TG, and CG), sexed them, weighed them, and released them at the capture location. Measurements across the longitudinal axis (HL, SVL, and TL) were done dorsally with a flexible sewing tape to the nearest 0.1 cm^34^ and circumference measurements were taken at the third tail segment (TG) and at the widest part of the chest (CG) and neck (NG) ^35^. Weight was measured with a variety of Pesola scales depending on the size of the animal to the nearest 0.1 kg for alligators under 50 kg and nearest 1 kg for alligators over 50 kg. We determined sex in most animals; however, some animals were too small (< 0.5 m) to accurately determine sex visually and few were not sexed. We removed alligators with incomplete tails in any analyses using TL to avoid biased allometric parameters.

We fitted log-transformed (natural logarithm) morphometric data with ordinary least squares (OLS) regressions via the *lm* function in R version 4.2.2^36^ to determine static allometric relationships between variables (isometry = 1.0 when evaluating unidimensional traits or 3.0 when evaluating mass traits, hyperallometry > 1.0 or 3.0, and hypoallometry < 1.0 or 3.0) assessing effects of sex, habitat, and sampling area in the slope and intercept (e.g., *lm*(*HL*–TL*Sex)). Allometric relationships were defined using only one decimal place so every relationship that falls between 0.96 and 1.04 was rounded to 1.0 (isometric). We used traits (HL, NG, CG, TG, SVL and W) as response variables and sizes (SVL and TL) as independent variables. Before defining final OLS models, we assessed morphometric relationships statistically for outliers via studentized deleted residuals (SDR) using the *studres* function from the “MASS” package^37^ and heteroscedasticity via the Spearman’s rank correlation test (*cor. test* function). Observations for which the SDR exceeded 3 were not included in the analysis, so that the final statistical model and regression parameters describes the dominant pattern in the data^38^. All regressions were graphed using “ggplot2” package^39^. We tested the levels of variability within models by covariates using an ANOVA and pairwise analysis comparing the estimated marginal means slope with *lstrends* function from “emmeans” package^40^. We adjusted confidence intervals and critical values (p-values) when simultaneous inference to control for family-wise error rate via Bonferroni correction using *lstrend*-*confint* and *test*-*pairs* functions, respectively. Statistical evidence was described based on p-values as very strong (< 0.001), strong (< 0.01), moderate (< 0.05), weak (< 0.10), or little-to-no evidence (> 0.10)^41^.

## Results

**Figure 1:**
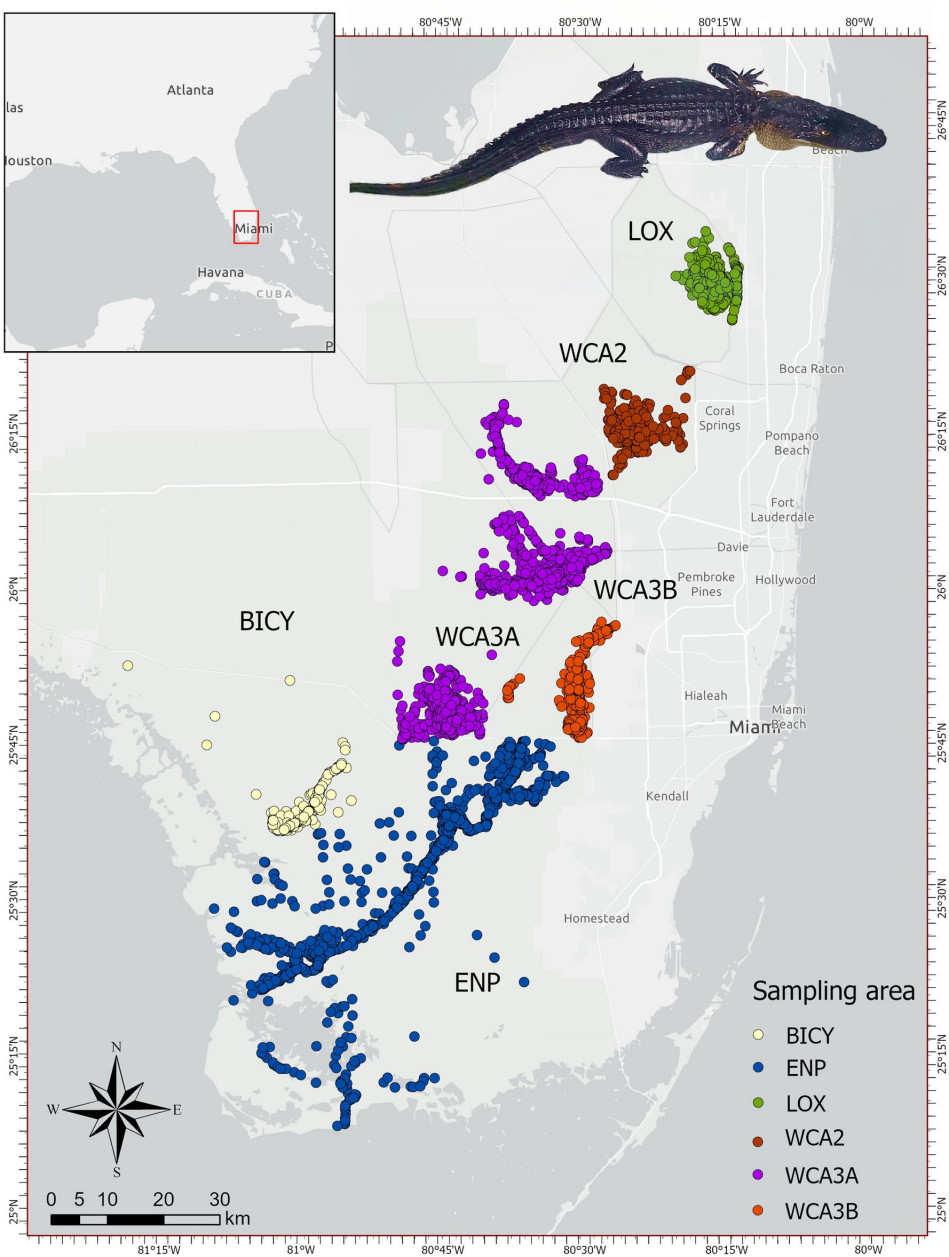
American alligator captured from 1999 to 2022 in the Greater Everglades, Florida, United States, by sampling areas—LOX (Arthur R. Marshall Loxahatchee National Wildlife Refuge), Water Conservation Area (WCA) 2, 3A, and 3B, BICY (Big Cypress National Park), and ENP (Everglades National Park). Insert: American alligator captured in WCA3A in 2018.

Alligators captured in this study ranged from 30.2 to 356.5 cm TL. However, 92% of the animals were above 125 cm TL (subadult and adult size classes) due to changes in survey protocols (target animal size) through time^33^. Alligators captured across the 22-year span of the study showed an almost even sex proportion with 0.96 females per 1 male (2670 females, 2771 males, 67 unknown). Most alligators were captured in marsh ecosystems (n = 4684) followed by rivers (n = 584) and manmade canals (n = 240). Across all sampling areas, most animals were captured within ENP (1867, 33.9%) and WCA3A (1655, 30.0%), followed by LOX (843, 15.3%), WCA3B (431, 7.8%), BICY (364, 6.6%), and WCA2 (348, 6.3%) (Fig. 1).

Regression models showed very strong evidence of a linear relationship between variables explaining equal to or above 92% of the variation in the data. Most of the trait–size relationships (8 out of 11 assessed) showed hyperallometry (positive allometry) with slope deviations from isometry between 0.1 and 0.2 units (Table 1). Slope variation (measured as 95% confidence intervals) was overall < 0.1 units across trait–size relationships showing high stability. In contrast, intercept variation varied across relationships with values as high as 0.32 for the CG–TL relationship and as low as 0.02 for the SVL–TL relationship. Only SVL–TL, HL–TL, and HL–SVL had an isometric behavior. On average, TL was approximately twice SVL (50.44 ± 1.08%) and sevenfold HL (13.87 ± 0.44%) and SVL was approximately fourfold HL (27.50 ± 0.78%).

**Table 1:** Ordinary least square linear regression parameters and statistics for the American alligator trait–size relationships captured in the Greater Everglades, Florida from 1999 to 2022. We found very strong evidence of linear relationships between all trait–size relationships (*p* value < 0.001) and no evidence of heteroscedasticity (*p* value > 0.1 ρ |0.05|).

Relationship	n	outliers	R^2^	ρ	Intercept (CI)	Slope (CI)
HL–TL	5141	36	0.99	0.05	− 2.09 (− 2.11; − 2.07)	1.02 (1.02; 1.03)
SVL–TL	5132	53	0.99	0.01	− 0.83 (− 0.84; − 0.82)	1.03 (1.03; 1.03)
TG–TL	5092	84	0.94	0.00	− 2.13 (− 2.17; − 2.09)	1.10 (1.09; 1.10)
CG–TL	462	4	0.92	0.02	− 2.33 (− 2.48; − 2.17)	1.17 (1.14; 1.20)
NG–TL	476	4	0.92	0.02	− 2.33 (− 2.48; 2.17)	1.13 (1.10; 1.16)
W–TL	5052	73	0.98	0.02	− 14.01 (− 14.07; − 13.94)	3.23 (3.22; 3.24)
HL–SVL	5422	32	0.99	0.06	− 1.26 (− 1.28; − 1.25)	0.99 (0.99; 1.00)
TG–SVL	5088	87	0.94	-0.01	− 1.22 (− 1.25; − 1.19)	1.06 (1.05; 1.07)
CG–SVL	461	4	0.93	0.00	− 1.29 (− 1.41; − 1.17)	1.12 (1.09; 1.14)
NG–SVL	476	4	0.93	0.03	− 1.33 (− 1.45; − 1.21)	1.08 (1.05; 1.11)
W–SVL	5316	82	0.98	0.01	− 11.34 (− 11.39; − 11.29)	3.13 (3.12; 3.14)

All data was log-transformed for analysis.

*HL* head length, *NG* neck girth, *CG* chest girth, *TG* tail girth, *SVL* snout-vent length, *TL* total length, *W* weight.

**Figure 2:**
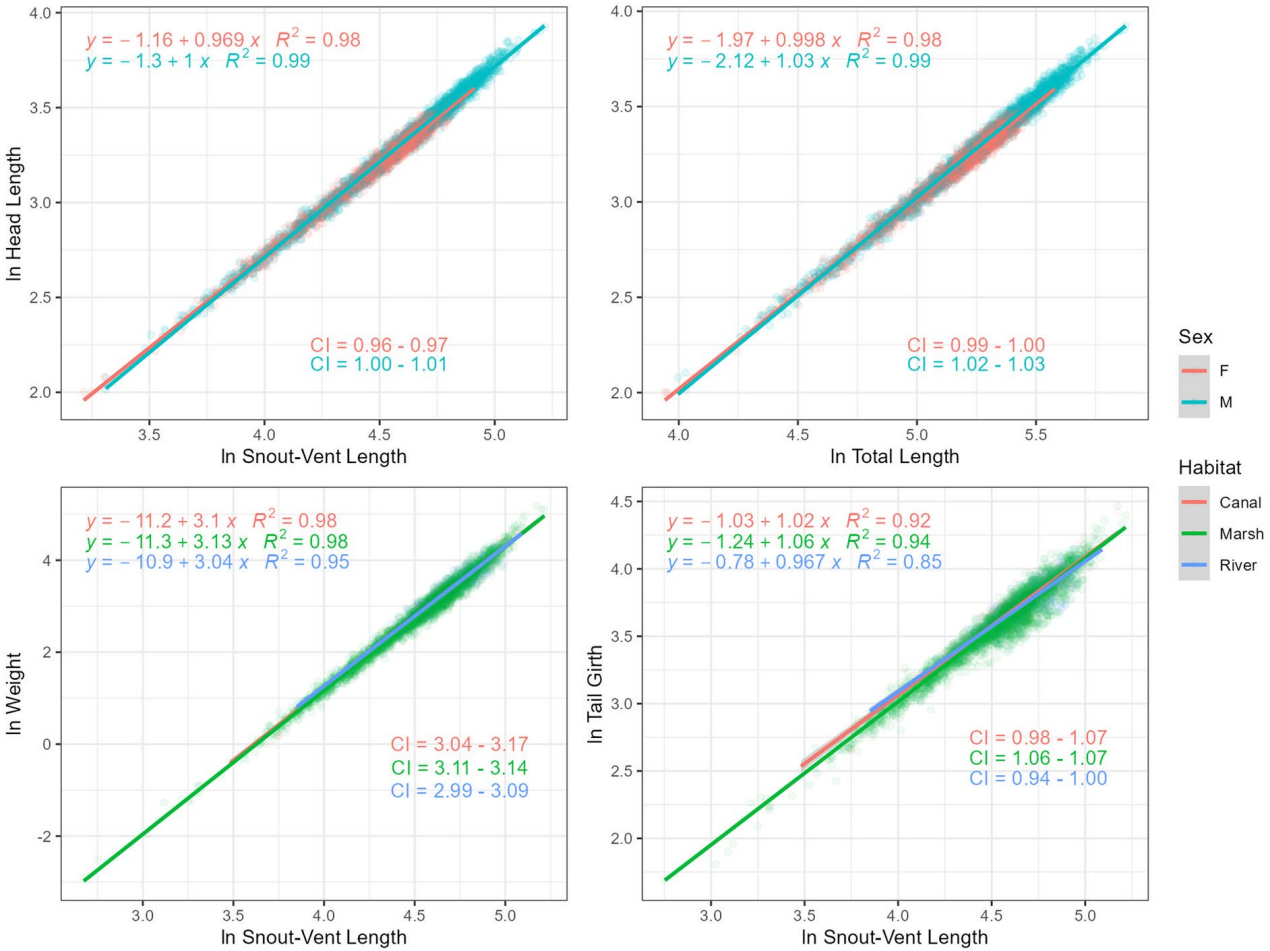
Trait–size ordinary least square regressions with evidence of an effect of sex and habitat on the slope. Regression parameters (intercept and slope), correlation coefficient (R^2^), and 95% confidence interval (CI) of the slope estimate are displayed on each graph. Outliers were identified and removed across all relationships prior to modeling. Statistics for these relationships can be found in Table 3.

Regression models showed no evidence of an effect of any of the variables assessed (sex, habitat, and sampling area) on traits such as CG and NG when related with either SVL or TL (Table 2). This pattern also holds true when assessing the effect of sex on most of the traits except when relating HL with either size (SVL or TL) where evidence of a strong effect [females having proportionally slightly lower no overlapping slopes (0.97 and 1.00) than males (1.00 and 1.03)] was found. Interestingly, intercepts showed an inverse pattern with males having lower values (− 1.3 and − 2.12) than females (− 1.16 and − 1.97; Table 3). This means that small size (SVL or TL) females have proportionally slightly larger heads than males but as alligators grow up males get proportionally slightly larger heads than females (Fig. 2).

**Table 2:** Analysis of variance (ANOVA) statistics derived from ordinary least square regression models.

Relationship	Degrees of freedom	Sum sq	Mean sq	F value	*p* value
HL–TL*Sex	1.000	0.070	0.070	70.165	**0.000**
HL–TL*Area	5.000	0.010	0.000	2.097	**0.063**
HL–TL*Habitat	2.000	0.000	0.000	1.534	0.216
SVL–TL*Sex	1.000	0.000	0.000	1.254	0.263
SVL–TL*Area	5.000	0.010	0.000	4.738	**0.000**
SVL–TL*Habitat	2.000	0.000	0.000	1.952	0.142
TG–TL*Sex	1.000	0.010	0.010	1.151	0.283
TG–TL*Area	5.000	0.200	0.040	8.634	**0.000**
TG–TL*Habitat	2.000	0.110	0.060	11.539	**0.000**
CG–TL*Sex	1.000	0.000	0.000	0.084	0.773
CG–TL*Area	3.000	0.022	0.007	1.436	0.232
CG–TL*Habitat	1.000	0.009	0.009	1.739	0.188
NG–TL*Sex	1.000	0.000	0.000	0.007	0.935
NG–TL*Area	3.000	0.002	0.001	0.144	0.934
NG–TL*Habitat	1.000	0.009	0.009	1.735	0.188
W–TL*Sex	1.000	0.000	0.000	1.852	0.174
W–TL*Area	5.000	0.300	0.100	5.258	**0.000**
W–TL*Habitat	2.000	0.000	0.000	0.430	0.651
HL–SVL*Sex	1.000	0.090	0.090	124.630	**0.000**
HL–SVL*Area	5.000	0.040	0.010	9.597	**0.000**
TG–SVL*Sex	1.000	0.000	0.000	0.184	0.668
TG–SVL*Area	5.000	0.270	0.050	10.895	**0.000**
TG–SVL*Habitat	2.000	0.180	0.090	17.798	**0.000**
CG–SVL*Sex	1.000	0.000	0.000	0.020	0.887
CG–SVL*Area	3.000	0.021	0.007	1.554	0.200
CG–SVL*Habitat	1.000	0.009	0.009	1.916	0.167
NG–SVL*Sex	1.000	0.000	0.000	0.004	0.953
NG–SVL*Area	3.000	0.003	0.001	0.216	0.885
NG–SVL*Habitat	1.000	0.010	0.010	2.105	0.148
W–SVL*Sex	1.000	0.000	0.000	0.233	0.630
W–SVL*Area	5.000	0.800	0.200	12.027	**0.000**
W–SVL*Habitat	2.000	0.100	0.100	5.575	**0.004**

This analysis assessed the levels of variability within a given regression model based on factoring covariate (sex, sampling area, or habitat). Bold numbers refer to relationships in which we got at least weak evidence (*p* value < 0.1) of an effect of covariates on the slope of the relationship.

*HL* head length, *NG* neck girth, *CG* chest girth, *TG* tail girth, *SVL* snout-vent length, *TL* total length, *W* weight, *Sum Sq* sum of squares, *Mean Sq* mean square.

**Table 3:** American alligator pairwise differences in slopes by sex, habitat, and sampling area across the present study.

Relationship	Contrast	Estimate	SE	DF	t ratio	*p* value
ln HL–ln TL	F-M	− 0.0304	0.0036	5109	− 8.38	0.000
	ENP–LOX	0.017	0.006	5129	2.763	0.086
	LOX–WCA3A	− 0.016	0.006	5129	− 2.779	0.082
ln SVL–ln TL	ENP–LOX	− 0.016	0.004	5120	− 4.140	0.005
	LOX–WCA3A	0.015	0.004	5120	3.910	0.001
	LOX–WCA3B	0.020	0.006	5120	3.600	0.005
ln TG–ln TL	BICY–LOX	0.072	0.019	5080	3.790	0.002
	ENP–WCA3A	− 0.040	0.010	5080	− 4.220	0.000
	LOX–WCA2	− 0.061	0.018	5080	− 3.450	0.009
	LOX–WCA3A	− 0.074	0.013	5080	− 5.770	0.000
ln W–ln TL	BICY–LOX	0.110	0.031	5040	3.590	0.005
	ENP–LOX	0.066	0.022	5040	3.010	0.039
	LOX–WCA2	− 0.105	0.029	5040	− 3.690	0.003
	LOX–WCA3A	− 0.096	0.021	5040	− 4.630	0.000
	LOX–WCA3B	− 0.093	0.031	5040	− 2.950	0.048
ln HL–ln SVL	F–M	− 0.034	0.003	5389	− 11.164	0.000
	BICY–LOX	0.035	0.007	5410	4.851	0.000
	ENP–LOX	0.030	0.005	5410	5.895	0.000
	LOX–WCA3A	− 0.030	0.005	5410	− 6.190	0.000
	LOX–WCA3B	− 0.029	0.008	5410	− 3.830	0.002
ln TG–ln SVL	BICY–LOX	0.082	0.019	5076	4.372	0.000
	ENP–LOX	0.046	0.013	5076	3.427	0.009
	ENP–WCA3A	− 0.0405	0.010	5076	− 4.231	0.000
	LOX–WCA2	− 0.0732	0.018	5076	− 4.170	0.000
	LOX–WCA3A	− 0.087	0.013	5076	− 6.815	0.000
	LOX–WCA3B	− 0.059	0.019	5076	− 3.036	0.036
ln W–ln SVL	Marsh–River	0.086	0.026	5310	3.306	0.003
	BICY–LOX	0.160	0.029	5304	5.454	0.000
	ENP–LOX	0.106	0.021	5304	5.149	0.000
	LOX–WCA2	− 0.132	0.027	5304	− 4.868	0.000
	LOX–WCA3A	− 0.139	0.020	5304	− 7.067	0.000
	LOX–WCA3B	− 0.167	0.030	5304	− 5.483	0.000

Notice that we present only relationships and contrast that showed at least weak evidence (*p* value < 0.10) of an effect.

*HL* head length, *TG* tail girth, *CG* chest girth, *SVL* snout vent length, *TL* Total length, W weight, *ln* natural logarithm, *LOX* Arthur R. Marshall Loxahatchee National Wildlife Refuge, *WCA* water conservation area, *BICY* Big Cypress Natural Reserve, *ENP* Everglades National Park, *SE* standard error, *DF* degrees of freedom.

Models including habitat showed very strong to strong evidence of an effect when relating W–SVL and TG–SVL. Further pairwise analysis showed that differences in slope by habitat for both relationships were due to marsh alligators having proportionally slightly higher (no overlapping) slopes (3.13 and 1.06) than river alligators (3.04 and 0.97). Interestingly, as in the case of sex, intercepts showed an inverse pattern with marsh alligators having proportionally slightly lower values (− 11.3 and − 1.24) than river alligators (− 10.9 and − 0.78). This means that small alligators (measured as SVL) living on river habitats have proportionally slightly higher weights and tail girths than alligators living on marsh habitats. However, larger alligators living on marsh habitats have proportionally slightly higher weights and tail girths than larger alligators living on river habitats. This trend is more noticeable in the TG–SVL than in the W–SVL relationship (Fig. 2).

**Figure 3:**
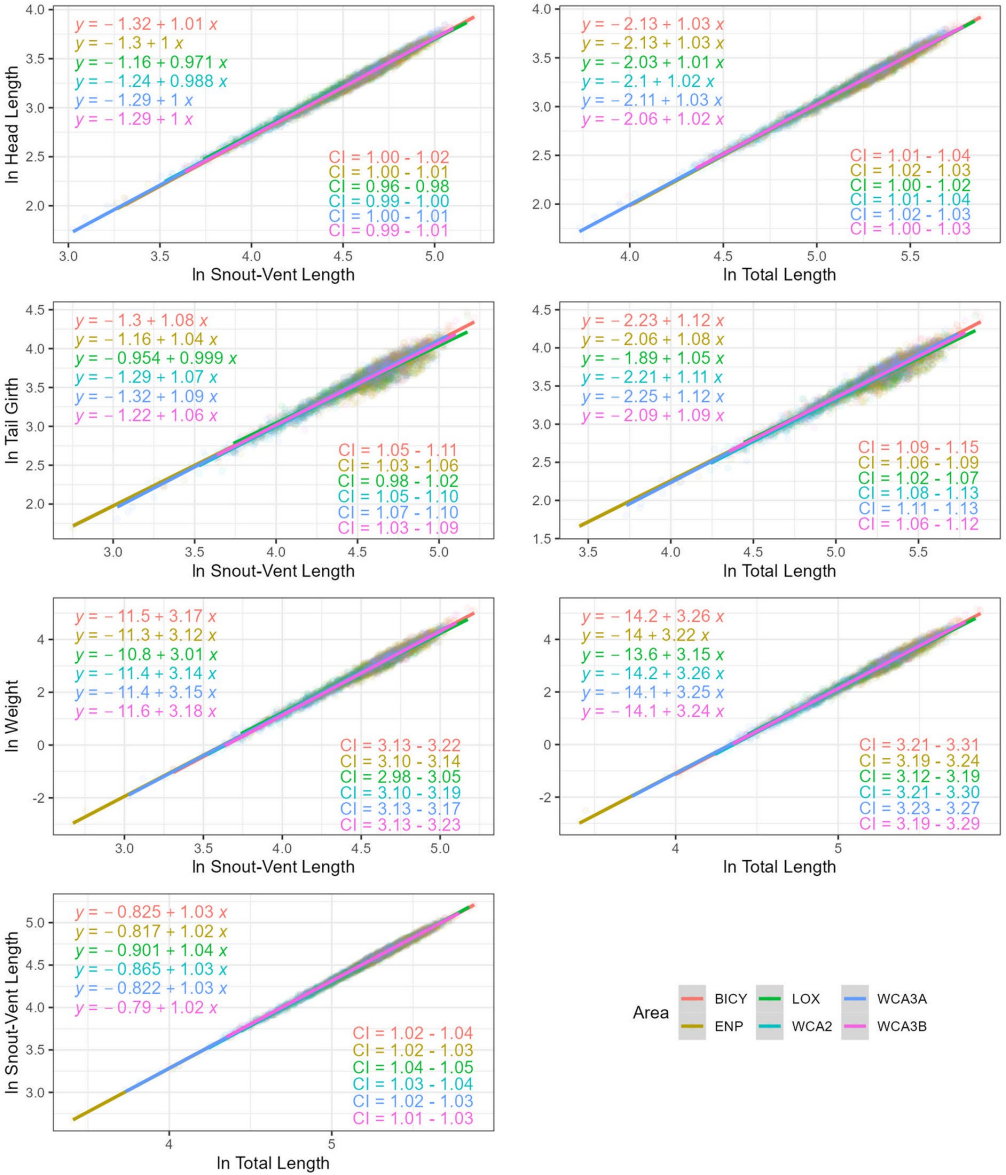
Trait–size ordinary least square regressions with evidence of an effect of sampling area on the slope. Regression parameters (intercept and slope), correlation coefficient (R^2^), and 95% confidence interval (CI) of the slope estimate are displayed on each graph. Outliers were identified and removed across all relationships prior to modeling. Statistics for these relationships can be found in Table 3.

Models including sampling area showed very strong to weak evidence of an effect when relating HL, TG, and W with SVL as well as HL, SVL, TG, and W with TL. Further pairwise comparisons showed that the slopes estimated in LOX differ from most of the slopes estimated in other sampling areas with little to no overlapping (Table 3). Specifically, we found that alligators in LOX are the most different ones in terms of slope across all areas showing lower values when compared to alligators captured in ENP and WCA3A based on HL–TL relationship (1.01 vs. 1.03 each), BICY, WCA2, and WCA3A based on TG–TL relationship (1.05 vs. 1.12, 1.11, and 1.12), and all areas (BICY, WCA2, WCA3A, WCA3B, and ENP) based on W–TL relationship (3.15 vs. 3.23, 3.22, 3.26, 3.25, and 3.24, respectively; Fig. 3). This pattern was also found when using SVL as size and related with all traits but increasing the number of sampling areas where slopes were different (all areas across all relationships; Table 3, Fig. 3). The only instance in which alligators captured in LOX showed higher slopes than alligators captured in any other sampling area (ENP, WCA3A, and WCA3B) was when relating SVL–TL (1.04 vs. 1.02, 1.03, and 1.02). Interestingly, we found an inverse pattern when analyzing intercepts with LOX having the highest values across most of the relationships except for SVL–TL in which it has the lowest value. This means that small size alligators captured in LOX have proportionally slightly higher tail girths and weights than in any other sampling area. However, larger alligators in LOX have proportionally lower tail girths and weights than alligators captured in other sampling areas. As in the case of habitat, this trend is more noticeable in TG–SVL than W–SVL and W–TL. Finally, the only two relationships in which we found evidence of an effect of sampling areas no related with LOX were TG–SVL and TG–TL between ENP and WCA3A.

## Discussion

Static morphometric allometry in alligators in the Greater Everglades showed a high tendency for hyperallometric relationships (positive allometry) and in a lesser extent isometry based on the six traits (HL, SVL, TG, CG, NG, W) and two sizes (SVL and TL) assessed in the present study. This mean that traits such as TG, CG, NG, and W became proportionally larger as individuals grows while traits such as HL and SVL grow proportionally to size through individual’s growth. This can be explained as former traits are related with fat-muscle gain—accumulation, which is influenced by prey quality—prey availability whereas grow and metabolic rate naturally slows down as alligators get older^42,43^, making it easier to gain fat—weight faster as animals get larger. In contrast, as reported by Dobson^25^, the HL–size isometry can be explained as the skull architectural factor in crocodylians is defined by the food-gathering function rather than the brain size factor (which is normally hypoallometric), which represent an adaptation to ever-increasing size of prey items as body size increases in generalist predators. These findings are in line with the isometric relationship found in spectacled caimans (*Caiman crocodilus*, slope = 0.95) and contrast with more specialized predators such as false gharials (*Tomistoma schlegelii*, slope = 0.78)^44^. Interestingly, several of the traits–size relationships assessed in our study were affected by sex (HL), habitat (TG and W), and sampling area (HL, SVL, TG, and W), which was unexpected (except for the weight trait) as allometry evolves very slowly (3% change in allometric coefficients per million years) and it requires a combined effect of multivariate constraints and stabilizing selection for changes to happen^2^.

Our study revealed two interesting extremes in relation to spatially static allometric relationships across the Greater Everglades for alligators. On one hand, alligators captured in LOX consistently showed lower slopes and higher intercepts across several traits (HL, TG, and W) with no overlapping confidence intervals (in the case of slopes) with any other sampling area, making it unique in shape, at least when relating these three traits with size. On the other hand, all other sampling areas showed a high variation in slope and intercept values specifically when evaluating TG (slope between 1.04 and 1.08 when related to SVL and between 1.08 and 1.12 when related to TL) and W (slope between 3.12 and 3.17 when related to SVL and between 3.22 and 3.26 when related to TL), showing a strong effect of sampling area on these two variables.

All these results have interesting repercussion not only in the way we see static allometric relationships in alligators as it is showing larger than expected variation in trait ~ size relationships that we presumed preserved across individuals (e.g. HL–size), but also in metrics such as body condition (either calculated from W–SVL or W–TL relationships) that are commonly assumed to be isometric and constant throughout space^8^. As an example, based on our results, smaller alligators in LOX may be proportionally heavier than smaller alligators captured in BICY but larger alligators in BICY are proportionally heavier than larger alligators captured in LOX (Fig. 3), likely representing differences in resource availability (prey) and habitat (marsh, river, canal) or habitat conditions (hydrology). The Greater Everglades has been exposed to human modifications for almost two centuries with large consequences in its hydrology and biodiversity^45^. These unnatural impacts have altered American alligator population dynamics affecting abundance, breeding cycles, landscape connectivity, and prey availability^11^, forcing the species to live in unfavorable conditions. We hypothesize that the high variation in static allometric coefficients in alligators found across the Great Everglades may be a reflection of alligator populations exposed to altered environmental pressures (e.g., hydrology). Further research assessing static allometric relationships in less impacted areas will allow to test this hypothesis and understand better the role of highly variable allometric coefficients in alligators.

Studies done on alligators in the last two decades in the Everglades have shown that population attributes such as abundance and body condition can be linked to changes in both hydrology and prey abundance—prey availability^8,9^. Our results confirm the usefulness of alligators as ecosystem indicators of hydrologic conditions and resource abundance as trait–size relationships such as W–SVL and W–TL can vary spatially depending on habitat conditions. However, it is highly relevant to first understand the behavior of trait–size relationships and their interaction with relevant variables (e.g., sex, sampling area) before using them to inform ecosystem management to avoid trait–size misinterpretations. Other trait–size combination that could also be used to inform the effect of sampling area on alligator body condition is TG–SVL as it also showed to be highly affected by this variable lining up with W–SVL results. If proved useful, this can reduce the stress of the animal as getting TG measurement is easier than weight especially in large animals (> 50 kg).

Sexual dimorphism has been constrained to total body length differentiation in alligators as well as most crocodylians (except gharials)^46^. However, our fundings suggest that there also can be sexual dimorphism in alligators when proportionally comparing HL with SVL or TL with small female alligators having proportionally slightly larger heads than males but as alligators grows up males get proportionally slightly larger heads than females. This is highly relevant as it is showing that the constant HL–size relationship no longer holds true and head extrapolations to total length can be off depending on sex. Our data show that this differentiation is more accentuated in small (< 4.5 lnTL, ~ 90 cm TL) and large (> 5 lnTL, ~ 148 cm TL) animals whereas intermediate sizes are more homogeneously distributed by sex, which means extrapolations done in middle size alligators will be more accurate than in small or larger animals. Total body length estimations from traits such as HL could be particularly useful when using unmanned vehicles (e.g., drones) as it is not always possible to observe the whole animal as well as when filtering out erroneous data or to obtain TL estimates when portions of an alligator’s tail are missing, especially in murky or densely vegetated habitat where only the head is observed^14^.

Although several studies have been conducted assessing morphometric relationships in crocodylians^14,15,17–23^, most of those studies have analyzed trait–size relationships arithmetically rather than geometrically (log-transforming raw measurements), which, as described in the introduction, increases bias and limits comparisons^24^. Biological variation is understood to be normally distributed based on the theoretical law of error, which has proven to differ by equal proportions (geometric) rather than by equal amounts (arithmetic)^47^. This means that, as stated by Gingerich^47^, arithmetic measurements must be transformed using logarithms to represent both the geometric normality of biological variation and the relative functional significance of measurements appropriately. Failing to do so can lead to misleading results that are not actually reflecting the nature of the relationship between the trait–size variables assessed. Thus, a revision of these allometric studies in a geometric context is warranted to really understand the allometry of crocodylia.

Allometric relationships reported in this study should be used to inform future research on population structure survey methods, body condition, as well as evolutionary biology in American alligators. Our work can be seen as setting the baseline for future comparisons within this population as more data is collected due to the restoration process going on in the area, but also among populations across species range (Texas, Louisiana, Alabama, Florida, Georgia, South and North Carolina). We recommend that alligator allometry studies are repeated across their range to compare allometric coefficients and allometric elevation as well as the effect of habitats and environmental parameters on allometric relationships. In all, understanding alligator allometric relationships is critical to determine patterns within their geographic range and to inform conservation and management of this important ecosystem engineer and keystone species.

## Data Availability

All data needed for the analysis performed in this manuscript is included in the manuscript. Raw data and code are available upon request.
